# Career Coaches as a Source of Vicarious Learning for Racial and Ethnic Minority PhD Students in the Biomedical Sciences: A Qualitative Study

**DOI:** 10.1371/journal.pone.0160038

**Published:** 2016-07-28

**Authors:** Simon N. Williams, Bhoomi K. Thakore, Richard McGee

**Affiliations:** 1 Department of Medical Social Sciences, Northwestern University, Chicago, Illinois, United States of America; 2 Feinberg School of Medicine, Northwestern University, Chicago, Illinois, United States of America; Charles P. Darby Children's Research Institute, 173 Ashley Avenue, Charleston, SC 29425, USA, UNITED STATES

## Abstract

**Introduction:**

Many recent mentoring initiatives have sought to help improve the proportion of underrepresented racial and ethnic minorities (URMs) in academic positions across the biomedical sciences. However, the intractable nature of the problem of underrepresentation suggests that many young scientists may require supplemental career development beyond what many mentors are able to offer. As an adjunct to traditional scientific mentoring, we created a novel academic career “coaching” intervention for PhD students in the biomedical sciences.

**Objective:**

To determine whether and how academic career coaches can provide effective career-development-related learning experiences for URM PhD students in the biomedical sciences. We focus specifically on *vicarious* learning experiences, where individuals learn indirectly through the experiences of others.

**Method:**

The intervention is being tested as part of a longitudinal randomized control trial (RCT). Here, we describe a nested qualitative study, using a framework approach to analyze data from a total of 48 semi-structured interviews from 24 URM PhD students (2 interviews per participant, 1 at baseline, 1 at 12-month follow-up) (16 female, 8 male; 11 Black, 12 Hispanic, 1 Native-American). We explored the role of the coach as a source of vicarious learning, in relation to the students’ goal of being future biomedical science faculty.

**Results:**

Coaches were resources through which most students in the study were able to learn vicariously about how to pursue, and succeed within, an academic career. Coaches were particularly useful in instances where students’ research mentors are unable to provide such vicarious learning opportunities, for example because the mentor is too busy to have career-related discussions with a student, or because they have, or value, a different type of academic career to the type the student hopes to achieve.

**Implications:**

Coaching can be an important way to address the lack of structured career development that students receive in their home training environment.

## Introduction

There has been much attention given to the very slow rates of improvement in the proportion of Black or African American, Hispanic or Latino/a, and Native American scientists, especially in academic positions and with NIH-level research funding [1–3]. This is clearly a multi-faceted problem beginning in early childhood and continuing throughout one’s professional training and experiences. However, one clear observation is the declining interest in and persistence toward academic careers during PhD and postdoctoral training [4, 5], particularly among these underrepresented racial and ethnic minority (URM) students [6]. Mentoring is one of the most critical determinants of a graduate student’s success and, for many URM students in particular, traditional mentoring may be inadequate [7]. Recent initiatives have sought to extend mentors’ training to include professional development competencies [8–11]. However, the intractable nature of the problem suggests that many students may require supplemental career development beyond what many mentors are able to offer due to the constraints they have in their availability to give time and training [12].

As an adjunct to traditional scientific mentoring, we created a novel ‘Academic Career Coaching’ (hereafter ‘coaching’) intervention for PhD students in the biomedical sciences, called The Academy for Future Science Faculty (‘The Academy’ for short). The design of this intervention and the research trial evaluating it has been reported in full in our study protocol [13]. In the Academy, established life scientists with demonstrated expertise in both diversity efforts and mentoring young scientists were recruited and provided additional training to help them in their role as ‘Academic Career Coaches’ (hereafter ‘coaches’) to a group of PhD students across in-person and virtual meetings. Although the intervention was designed to provide career development activities to students across all racial and ethnic groups, a particular objective was to provide these resources to URM students. The design of the intervention was novel in that students were split roughly equally across racial/ethnic and gender groups, and were drawn from a variety of biomedical disciplines and institutions across the United States. Coaches also came from different institutions than their coaching groups. The main ways in which coaching was designed to supplement traditional research mentoring is in Table 1.

**Table 1:** Key differences between traditional research mentoring and Career Coaching.

	Research Mentors	Career Coaches
*Career Guidance/Professional Development Training*	Receive infrequent or idiosyncratic formal training on how to guide students’ careers and professional development	Receive formal, intensive training, grounded in social science theory, on how to guide students’ careers
*Diversity Training*	Very unlikely to receive effective diversity training from institution	Receive intensive diversity training, informed by social science theory, focused on issues of “being different”, and potential isolation or discrimination.
*Independence*	Mentors usually from same institution as students. May be competing demands on mentors between their role as principal investigator and their role helping to advance students’ careers.	Coaches drawn from different institution to students. Are able to provide an outside perspective on career development.
*Groups As Well As Individuals*	Mentors usually work only with individuals one at a time. Unable to draw on strengths and efficiency of guiding peer groups.	Coaches provided training in working with groups, are able to use teaching techniques possible in groups, and activate peer support and learning. Groups can provide safe environment to talk about difficult topics.

The design of the Academy and its activities were also unique in that they were informed by social science theories and concepts [13]. One of these concepts was self-efficacy [14], and one of the main objectives of the Academy was to provide an environment within which URM students’ self-efficacy toward their goal of being future biomedical faculty could be positively impacted. One of the major components of self-efficacy is vicarious learning. In this paper we focus on URM students in the Academy group, and discuss the role of the coach as a source of vicarious learning. Using a qualitative approach, we explore the vicarious learning experiences of URM PhD students in the Academy specifically related to their goal of being future faculty. The findings of this paper are split into two parts: 1. Pre-intervention (baseline) and; 2. At 12 months follow-up. Over this 12-month period, students participated in an intensive two-day in person meeting, where they received intensive coaching and engaged in a variety of group discussions and interactive lectures and workshops. They also were thereafter encouraged to communicate with their coaches via regular one-on-one telephone conversations and/or online video-conferences with their coaching group, as well as via email and social media (see methodology section, or study protocol [13] for more details).

The main aim of this paper is to determine whether and how the coaches in the Academy can provide effective career-development-related vicarious learning experiences for URM PhD students in the biomedical sciences. We address this question in the second part of our findings. Before we can do so however, it is first important to explore whether and how these students are getting or lacking career-development-related vicarious learning experiences from their research mentors in their home institutions.

### Vicarious Learning

Self-efficacy is defined as an individual’s beliefs about their capabilities to organize and execute courses of action required for attaining designated types of performances [14, 15]. Beyond one’s own past achievements, or ‘personal performance accomplishments’, one of the most important ways in which self-efficacy is influenced is through ‘vicarious learning’ [14, 15].

‘Social persuasions’ (e.g. receiving constructive feedback) and ‘physiological and affective states’ (e.g. performance anxiety) also influence an individual’s self-efficacy, but are not discussed in the current paper. Vicarious learning, or ‘modeling’, is where ‘an observer learns from the behavior and consequences experienced by a model rather than from outcomes stemming from his or her own performance attempts’ [16]. Outcome expectations are an individual’s personal beliefs about the consequences or outcomes of performing particular behaviors (i.e. ‘if I do this what will happen?’) [17]. Research has shown how amongst PhD students, vicarious learning experiences play a strong role in shaping outcome expectations about the process of obtaining a faculty position [7]. Race and ethnicity, as well as other personal and contextual variables (e.g. gender) can impact an individual’s career interest, choice, and performance via the differential learning experiences, including vicarious learning experiences that give rise to the individual’s self-efficacy beliefs about that career [17]. Vicarious learning experiences are particularly important in instances where an individual has not yet been able to develop personal mastery, for example, a PhD student who has not yet had direct experience of being an academic faculty member. As well as from directly observing behavior (e.g. watching someone give a job talk), vicarious learning can also be derived from ‘verbal modeling’ and the ‘second-hand information’ one acquires about a field [18–20] (e.g. hearing about another’s career experiences). Additionally, more complex and problem-solving forms of learning rely on more ‘abstract’ modeling, where ‘the ordinarily covert thoughts guiding the actions of the models are made observable and learnable by others’ [21]. Such vicarious experiences can demonstrate how the social rules associated with a particular behavior or role can be applied or adjusted to fit the learner’s circumstances [14]. As such, vicarious learning results in the conversion of modeled activities into representational guides for future action [22]. Students who observe a successful model in a specific career field are more likely to develop a preference for pursuing that career and are more likely to believe they could be successful in that career [23]. Thus modeling, including verbal modeling can build self-efficacy [24]. Successful models provide important vicarious learning experiences, and this may be particularly so where the model has a similar cultural identity [25].

## Materials and Methods

The Academy for Future Science Faculty study was reviewed and approved by Northwestern University’s Institutional Review Board, Project STU00035424. Prior to participation in the study, participants provided full written informed consent.

### Setting and Design

The Academy is a large-scale intervention testing the impact of career coaching on biomedical science PhD students’ interest in, and persistence into, academic careers. In particular, the intervention aims to positively impact the academic career development of URM students. The Academy has two cohorts running concurrently: Cohort 1, which recruited beginning-stage PhD students (about to enter their 1st year), and Cohort 2, which recruited late-stage PhD students (<18 months from completion). In this paper, we analyze and discuss the perceptions of URM students in Cohort 2. Additional details and discussion about the intervention can also be found in previous publications [13, 26].

To explore students’ perceptions of their experiences in the Academy, we have been conducting annual interviews. This study incorporates interview data at baseline (<2 months prior to the start of the intervention, June—July 2012) and after 12 months of the intervention (June—July 2013). The aim of this paper is to provide an in-depth qualitative analysis, informed by the analytical concept of vicarious learning, of the ways in which coaching worked to maintain URM students’ interest in academic careers, thus enhancing their academic faculty career self-efficacy. All research related to the Academy was reviewed and accepted by Northwestern University Institutional Review Board, Project STU00035424.

Although the bulk of existing literature on self-efficacy takes a quantitative approach, it has been argued that qualitative interviews offer a phenomenological lens through which the development of efficacy beliefs can be viewed, and enables researchers to examine the different conditions under which students’ process and appraise their experiences at particular junctures in their education [27, 28]. Additionally, interviews provide useful insights into students’ perceptions of their self-efficacy in relation to contextual challenges and barriers (e.g. lack of career guidance or support) [29].

### Participants

In summer 2012, 60 advanced-stage biomedical PhD students were recruited to take part in the Academy Cohort 2 trial. Of these 60 students, 32 are URMs. Eligibility criteria for this arm of the study were: (a) enrolled in a U.S. biomedical PhD program, (b) expressed interest in an academic career, (c) US citizenship or legal permanent residence, (d) be within approximately 18 months of PhD completion. Random stratified sampling was used to ensure that the Academy contained roughly equal numbers of students from each gender and from each racial/ethnic group. Students were then sub-divided into six ‘coaching groups’ of ten students each. Allocation to each coaching group was also stratified such that no racial/ethnicity or gender was a majority (i.e. each group had 4–6 females and 4–6 males, including 2 Asian, 2 Black, 2 White, and 2 Hispanic, with the remaining students, including 2 Native American students, randomly allocated across the groups). Additional details of the full study population of the intervention–not just the URM students discussed in this paper–can be found in the study protocol [13]. Of the 32 URM students taking part in the intervention, 24 provided interview data both pre-intervention and at 1-year follow up, and are subsequently included in this analysis. Table 2 displays the characteristics of the population discussed in this paper.

**Table 2:** Summary characteristics of Participants discussed in the present analysis.

*Participant Characteristic*	*N (%)*
*Gender*	
Female	16 (67)
Male	8 (33)
*Race/Ethnicity*	
Black	11 (46)
Hispanic	12 (50)
Native American	1 (4)

Additionally, six coaches were recruited among leaders of research training and diversity efforts in U.S. universities. Announcements were made through program and organization lists or listservs. Coaches were given additional training during an initial 2-day meeting and in subsequent remote conferences. A key element of the coaches’ training included a workshop on how social science concepts, including self-efficacy, can be used to help interpret some of the social and psychological processes that attract or detract young scientists, particularly URM students, toward or away from academic careers.

### The Intervention

Coaching in the Academy included annual, intensive two-day in-person meetings held in Chicago, supplemented by year-round distance communication. By the time of their second interview in 2013, students had participated in one 2-day in-person meeting and had approximately one full year of contact with their coach and coaching group. The in-person meeting included coach-led presentations and panels for the intervention group as a whole, and coach-facilitated activities in individual coaching groups. For example, guided by their coach, students completed and then discussed Individual Development Plans (IDPs) and self-assessment documents. Over the 12 months following the in-person meeting, students were in contact with their coach an average of 9 times (range: 2–25; SD: 5.4) via one-on-one calls, coaching group video-conferences and email or social media. Although the research team provided suggestions on how to structure these virtual group meetings, coaches and coaching groups were encouraged to address any issues they deemed relevant to professional and personal advancement in real time. The number and content of virtual coaching group meetings, and numbers of individual student-coach contacts, varied substantially between groups. Thus, this analysis does not compare groups but rather looks at impacts on self-efficacy when they occurred.

The Academy sought to positively impact students’ self-efficacy concerning their professional development in graduate school and personal goals of becoming future science faculty. Coaching group peers were also promoted as potential sources of vicarious learning, and early evidence suggests that students may have benefitted from their peers in this way. The influence of coaching group peers will be discussed in subsequent papers. In this paper, we focus exclusively on the role of the coach and explore how they provided opportunities for vicarious learning related to students’ career development and goals.

### Data Collection

Forty-eight interviews (2 per participant), each lasting between 45 minutes and 1 hour 20 minutes, were conducted via telephone by members of the research team, all of who had professional experience in qualitative interviewing. As noted, participants were interviewed just prior to the intervention, and after they had received approximately 12 months of coaching. Both interviews followed semi-structured interview guides covering a range of topics related to participants’ career goals and experiences in graduate school. In this paper, we focus on responses from the first interview to questions exploring students’ relationships with their research mentors and the career-related knowledge they had received thus far. From the second interview, we focus on responses to questions exploring students’ relationships with their coaches and the extent to which the coach was perceived as being effective or useful.

### Data Analysis

Interviews were professionally transcribed and were secondarily checked and edited by a member of the research team to ensure accuracy. Interview transcripts were analyzed using the framework approach [30, 31], in which qualitative data are coded and organized according to themes and sub-themes, and data matrices are used to facilitate analysis. One author (SW) led the analysis, while a second author (BT) contributed to the analysis. Both authors regularly met to discuss and consult with each other during the course of the analysis, as well as to consult with the third author and principal investigator of the overall study (RM), and additional members of the research team. This framework approach entailed a number of processes. All authors familiarized themselves with the interview by briefly reading and discussing interview transcripts. We developed an analytical framework for the analysis and two authors (SW and BT) coded a sample of transcripts. The framework approach can be primarily deductive or inductive depending on the particular research question [30]. As noted above, the primary aim of the overall study was to evaluate the effectiveness of the coaching intervention, and the specific aim in this paper was to explore whether coaching impacted URM students’ self-efficacy by providing vicarious learning experiences. As such, there was a strong deductive component to our analysis. Our framework drew on the concept of vicarious learning, building on relevant literature related to Social Cognitive Career Theory (SCCT) [18], as well as Social Cognitive Theory (SCT) more broadly [14]. We looked both for evidence for and against out hypothesis that coaches would provide vicarious learning experiences. However, coding also allowed for themes to inductively emerge, both to help define and expand the concepts of vicarious learning in relation to the specific context of our study, and to generally accommodate any novel and unanticipated findings. Authors discussed initial data at various points during the analysis process and initial coding was discussed in order to check consistency and to help finalize the analytical framework. One author (SW) then used this final coding framework to code the remainder of the transcripts. The data was charted into a matrix so that prominent themes could be visualized. Interpretation was an ongoing process, with data discussed at various points during the process in research team meetings. The interpretation of our data is presented below, with illustrative quotations used to exemplify themes. Pseudonyms are used throughout to ensure participants’ anonymity.

## Results

### Findings from baseline interviews: Students’ relationships with research mentors

In their baseline interviews, participants were asked to describe their relationship with their mentors from their PhD institution. In particular, students were encouraged to discuss this relationship in terms of its usefulness to their career development and to their goal of an academic career.

10 participants (42%; 3 male, 7 female) felt that one or more elements of their relationships with mentors was ‘limited’ (Alfred, Hispanic Male) or that they ‘hadn’t gotten what they needed’ (Tanesha, Black Female) or were ‘missing out’ on a certain amount of stuff’ (Charlene, Hispanic Female). 14 participants (58%; 5 male, 9 female) felt that they had ‘gotten the mentoring [they] need during grad school’ (Frank, Black Male) or had ‘gotten enough’ (Blanca, Hispanic Female) mentoring, including career-related mentoring.

#### Students’ career-development-related vicarious learning experiences with research mentors

Of those who were felt that they hadn’t gotten what they needed from their mentors, one of the main reasons for this was their mentors’ general unavailability. In order to learn vicariously from models, students need to be able to have adequate access to, and interactions with, them. However, many students’ research mentors often have numerous competing demands on their time (related to their own research for example), meaning they have little time available for having career-related discussions with their students. As Alec explained:

I want them [mentors] to have an open door where I can just go sit down and talk with them for 20 minutes if I need to, every day, or once a week, or whatever it may be. But right now it’s tough to track my advisor down because he’s so busy (Alec, Hispanic Male).

By not having, or making, the time for such discussions, students are unable to learn about their mentors’ own career paths and experiences.

Of those who felt that they had gotten what they needed from their mentors, including career mentoring, two participants also acknowledged that their mentors were busy, but felt that it was important to be independent and proactive in guiding their own careers. The remaining 12 participants tended to describe in very positive terms how they had been able benefit from a positive or supportive relationship which often enabled them to “learn about the process of being in academia as well as being a scientist” (Jefferson, Black Male). These students tended to identify with their mentors and had been able to learn vicariously from their experiences:

I have mentors who are older than me … it’s identifying with them that grad school is very tough and the transition from grad school to postdoc is also tough. But if you can get an idea of what to expect, that’s great … It’s just getting to know their experiences by talking to them (Jacqueline, Native American Female).

Jacqueline’s quote exemplifies the benefits of having more advanced models who are able to convey their own past experiences as a means of demystifying the steps to a faculty career—for example, transitioning into a postdoctoral position.

Participants were also asked what they hoped to get from their Academy coach. All participants suggested they were hoping to learn more about how to successfully pursue an academic career, though responses varied as to exactly the types of issues they sought to better understand. Some had specific issues or questions they wanted to discuss or learn more about. For example, Jacqueline wanted to learn more about how to balance research with teaching. As she said, “I think that I want to learn the logistics of running a lab and balancing teaching with it. I want to have someone who has a passion for teaching but also being in the lab.”

Students also tended to discuss how they looked forward to receiving advice from someone who was not in their home institution:

I’m looking forward to tapping in[to] someone else who is not in my university … Are their experiences the same as the ones that I have had here? And [I’ll] kind of compare and understand more what it is that I’ll be exposed to in the future (Blanca, Hispanic Female)

As Blanca’s example suggests, participants hoped to be able to use the coaches’ experiences to help clarify their expectations about the future. Blanca was aware that the lab, department or institution she would be in as a postdoc might be different to the lab, department or institution she had been in during graduate school. As such, she hoped that she could learn vicariously from her coach’s experiences.

#### Students’ perceptions of the role of race-ethnicity and their relationships with research mentors

Participants also discussed their career development and their relationships with mentors in regard to the issue of race, ethnicity and underrepresentation in science. In so doing, some described feeling ‘handicapped’ as a result of not having access to models they could identify with, within their family, school or department:

I don't know if it’s a race thing or just being first generation but it’s always harder when you're like the trail blazer. You're the one who’s learning things when other people haven't done it before. So I always feel like I have really no concept of [the] general rules of what it took to become a PI. I would just try to be learning as I'm hearing from other people who like either have parents or know someone who did that [become a PI]. … so I felt like, I guess, a little bit handicapped or slighted that I haven’t had this knowledge (Dale, Hispanic Male).

Like many URM students, Dale saw himself as having to be a ‘trail blazer’ due to the lack of models from which they could learn how to prepare themselves (“build your CV”) for a faculty career. In the sample discussed in this paper, only 6 students (25%) had at least one parent that had completed graduate school (with either Master’s degree or a PhD) and 9 students (38%) were the first generation in their family to graduate with a Bachelor’s degree. Dale’s account exemplifies the importance of vicarious learning as a means to understand the unwritten ‘rules’ of succeeding in a faculty career. As noted above, vicarious learning, particularly where it pertains to complex domains—for example, those associated with career and professional development–can help to demonstrate how the social rules governing that domain can be made ‘learnable’ and can therefore be applied and adjusted accordingly [14, 21]. Others also discussed the importance of interacting with, or even simply being aware of, successful URM faculty:

I don't see many [Latinos] like me. It's a fact that there is unequal representation in academia and so I don't see very many Latino faculty. … Not seeing as many can sometimes cause some insecurities like ‘wow, can I make it then or am I going to have a tougher time making it?’ The support isn't as visual or the goal isn't as visual (Alfred, Hispanic Male).

Alfred explains how not observing Latino faculty has the negative impact of making his goal of an academic career seem less achievable or less ‘visual’. As established, an individual’s perception of the environmental barriers that he or she faces (e.g. related to their race/ethnicity) shapes the course of their career development [29].

Overall, 13 participants (54%) felt that it was important to have mentors with life experiences similar to their own, including experiences pertaining to race and ethnicity. Three participants (12.5%) felt that this was not important and that they would rather prefer to have mentors with different such life experiences. Another three participants (12.5%) thought that it could be beneficial in certain respects or instances. As Aurora (Black Female) stated, “I think it can be useful but is not necessary all the time”. The remaining five participants (21%) did not discuss whether or not having access to URM faculty was important to them. Twelve participants (50%) had previously or currently had a mentor that was of the same racial or ethnic background as them during college or graduate school. However, there was no pattern as to whether or not those who had a same-race/ethnicity mentor were more or less likely to feel it was important or beneficial.

Amongst those students who felt it was important to have mentors with similar life experiences to them, nine were female participants (56% of the 16 women) and four were male participants (50% of the 8 men). One particularly interesting finding that emerged was that four of the female students discussed how it was beneficial to have a mentor that understood being a minority and a woman in science, citing the added challenges related to the intersection of gender- and race-based discrimination. URM males however were less inclined to discuss their gender as a reason as to why it was beneficial to have a mentor of similar life experiences, instead focusing mostly on race or ethnicity. URM females however were more likely to explicitly discuss experiencing incompatibilities with their research mentors, where these mentors were of a different race, ethnicity and gender. For example, Ebony, whose mentor was a white male, discussed how her PI made her feel as though she didn’t fit the ‘mold’ of a scientist.

I think he wants to be a good mentor, but doesn’t know how to do it … I think I would like to balance teaching and research [and] I kind of feel like being a scientist is a public service and I would like my research to be relatable to the layperson … I think he perceives me as not being tough enough [to be a successful scientist] but I have been through many hardships in my life … In his mind he has a vision of what a scientist is like and an older Caucasian man is his perception of who is a successful PI. … I feel like this has nothing to do with science … He looks for people that emulate himself … I feel that he feels I need more molding than the other grad students (Ebony, Black Female).

Ebony describes how her mentor had failed to understand or relate to some of her life experiences and had failed to appreciate her career goals. Unlike her research mentor, who was primarily focused on research, Ebony combined her research interests with a passion for teaching and for science outreach and communication, and had worked with community initiatives trying to broaden participation in science in young and economically disadvantages minority populations. Ebony also described how her incompatibility with her research mentor was related to the latter’s stereotypical perceptions of the typical ‘successful PI’ as a majority male scientist.

Like Ebony, Lacey, also described the challenges of not looking like a ‘typical’ scientist:

When they think of a scientist, they think of somebody who's wearing glasses, with a white lab coat on, and they're white, and probably a white male…. I don’t know if I’ve had somebody that stands out in my mind as a mentor. It's difficult to just make up those relationships. … I would say it probably is helpful to get someone—especially if they are guiding you along the career journey—that [has] gone through your experiences … But the minority faculty always has so many other things and pressures already going on (Lacey, Black Female).

In this extract, Lacey also alludes to the fact that URM faculty often assume additional roles and pressures associated with being (usually) one of the few persons of color in a department or institution–for example sitting on diversity-related committees. Thus, even where potential URM models exist, they are seen to be as busy, if not busier, than their non-URM counterparts when it comes to being available to provide substantial and experience-based career development to their mentees.

Rosemary also described what she felt was the negative effect of her colleagues’ unconscious biases:

I used to think I was talking to scientists who were my peers and we were just talking science, but the racism and sexism in this country is so thick when they’re talking to me, they’re looking at me first as a black person, maybe as a Hispanic as I’m both and then as a female … they’re entrapped by their own biases. So I have to get through all those filters before I can start talking science. … A lot of white men do not want to train people of color. They feel very competitive and very insecure about someone else learning what they know (Rosemarie, Black and Hispanic Female).

Grounded in her own lived experience, Rosemarie argues that relationships between white male mentors and URM female students can sometimes be problematic because these unconscious biases and the mentors’ competitive attitude act as a barrier to the students’ vicarious learning.

The three participants that felt it was not important to have mentors with similar life experiences tended to emphasize the importance of hearing a diverse range of perspectives, including those of others with different as well as similar life experiences, including racial-ethnic backgrounds:

I actually like hearing people both from what I do but I also do enjoy people who have different experiences because it kind of just gives me more of an outlook of how other people do things and how they incorporate things (Dale, Hispanic Male).

### Findings from follow-up interviews: Students’ relationships with coaches

In their follow-up interviews, participants were asked to describe their relationship with their coach. Specifically, participants discussed whether their coach had been effective. Seventeen participants (71%) felt that their relationship with their coach had been effective. This included nine out of the ten students (90%) who, in their first interviews, felt they were not getting what they needed from their mentors. These students tended to feel as though the they learned a lot from the coaches during the in-person meeting, and tended to interact with their coach quite frequently over the 12 months (11 times on average, ranging from 3–25 interactions over 12 months).

Five participants (21%) felt that the coaching they had received during the in-person meeting was generally effective, but that they had not had much subsequent interaction with their coach remotely over the 12 months (4 times on average). This included four of the 14 students who had previously felt as though they had gotten what they needed from their mentors. Finally, two participants (8%) felt as though the coaching they received was not effective. Both students had in their first interviews described feeling as though they had gotten what they needed from their mentors, including career mentoring. These students, who were both allocated the same coach, tended to feel as though their coach had not been very effective for them in-person and they had also not had much remote interaction with them over the 12 months (interacting on only 2 and 5 occasions respectively).

#### Students’ career-related vicarious learning experiences with coaches

As we saw from their first interviews, those participants who felt like they were not getting what they needed from their mentors, including their career mentoring, tended to feel as though their research mentors were too busy to dedicate enough time to this topic. The coaches’ main purpose, however, was to provide dedicated time for career-related discussions. This was a prominent theme in participants’ second interviews. For example, Alec, who as noted above, wanted more time with his mentor who was “so busy”, was able to have new types of conversations with his coach:

He made it very clear that he was open to us contacting him … So I expected that he would be available if I emailed him … He also made it clear that he would be having monthly video teleconferences … [During] the face-to-face meeting he was very open and honest with us and I think he made us all feel comfortable talking about difficult situations. In one instance he talked about one of the most difficult situations that he’s experienced in his life when he was denied tenure at [University]. … That sort of conversation is not something I really have with research mentors (Alec, Hispanic Male).

Unlike his research mentor, Alec’s coach was able to establish dedicated time for career development, and made clear his availability for such discussions. For Alec, hearing about his coach’s experiences–for example, initially failing to get tenure–was a source of vicarious learning about the types of challenges that might be encountered on the path to a faculty career. Because his coach was now successfully tenured at a different university, it served as a positive model for how such difficulties can be overcome.

Eight students who had felt that they had generally gotten what they needed from their mentors described having an active and effective relationship with their coach. For example, Jacqueline (Native American Female) who, as we saw in her first interview, was able to identify with more experienced mentors, including her current PI, and “get to know their experiences”. However, in her second interview, Jacqueline discussed how having a coach was still “a positive experience” because she was able to learn from the experiences of her coach, whose academic career path was more akin to the specific type of academic career that she herself desired. She still characterized her relationship with her PI as being a positive one, but reflected on how her career plans sometimes conflicted with her PI’s plans for her:

“My PI and I get along pretty well. I think the only thing was just that we weren’t sure what would be the best next step. I was thinking one thing and she was thinking another. I have received so much help from her, but at the same time, I feel that I get to choose what I do next. … I think it’s not a good fit for me by having too much of a research demand … I’m more into the idea of teaching but I still want to do some research on the side. I think every department is different, [and] every institution is different. … Right now I think my next step of having the teaching-research post doc would help me build up my understanding … [At] the meetings with him [my coach] at the Academy … I was able to really get to learn about how he got to where he was and his experiences going through the post doc search. Because I was torn about maybe just getting an academic job right after finishing [the PhD] or doing a post doc, what type of postdoc [to do]. He helped me learn about the IRACDA [Institutional Research and Academic Career Development Awards] program. He [also] helped me learn about how he felt as a PI [and] how he’s had more of an impact on the teaching side of young scientists” (Jacqueline, Native American Female).

This extract exemplifies a common concern, namely that students’ PIs were either inclined to guide them towards one specific type of academic career (e.g. a research-intensive career) that was favored by the PI but not necessarily by the student. In such instances, the PIs did not necessarily function as effective models because the student desired a different type of academic career than the PI’s. In these instances, having a coach was beneficial for two reasons. Firstly, it provided the students with new experiences and insights, and many of the coaches had experience across a range of academic skills and roles, including teaching. In this particular example, Jacqueline’s coach had considerable teaching experience as well as research experience, and had been involved in the IRACDA program–a program seeking to provide teaching experience to young scientists–and was thus served as a new source of vicarious learning. Secondly, the coaches intentionally avoided placing emphasis or assigning importance to any particular type of career (including academic and non-academic science careers). This gave the students more freedom to discuss different types of potential career trajectory.

The five students who felt that they had learned from their coaches during the in-person meeting but had not interacted much with them during the 12 months, tended to emphasize the fact that they were also themselves”busy” with their own research “so didn’t have a need to reach out” (Jamie, Hispanic Male). The Academy was designed to counsel coaches to be available but to not push a higher level of engagement than students’ desired.

Another prominent theme in the second interviews was that, irrespective of the quality of mentoring or career guidance they had received in their home institution, many students felt they derived a “benefit of having somebody who’s not necessarily affiliated with my institution” (Wilson, Black Male). Blanca, as we saw in her first interview, was looking forward to tapping into her coach’s experiences. After a year of the intervention, Blanca had formed a close relationship with her coach, referring to her as a “role model” from whose experiences she could learn vicariously:

I would say that actually the coach that was assigned to us, was one major source of guidance outside of [name of student’s home institution]…whatever issues have come up she [the coach] would comment on it and bring her own experience, and there would be some times that I had never even have thought about looking at that issue the way that she kind of described, so I do consider her a role model (Blanca, Hispanic Female).

For students like Blanca, hearing about the coaches’ experiences encouraged new perspectives and new ways of looking at some of the challenges they faced during graduate school and in relation to their career planning.

The two students, who shared the same coach, that felt as though the coaching they had received had been ineffective, tended to describe that there were “not getting something from it” (Aurora, Black Female). For them, their coach also appeared to be too busy to provide adequate career guidance: “I know that [the coach] is probably busy, but just staying on top of things would have been more little more beneficial or productive” (Aurora, Black Female).

### Students’ perceptions of the role of race-ethnicity and their relationships with their coaches

As we saw from their first interviews, participants held a variety of perspectives as to whether or not it was important having mentors with similar life experiences and racial and ethnic backgrounds. Overall, we did not find any major difference between URM and non-URM coaches in terms of whether or not participants perceived them to be effective. That is, in their second interviews, participants were just as likely to describe non-URM coaches as useful sources of vicarious learning as they were URM coaches.

Of the 12 participants in the sample that had a non-URM coach, eight saw their relationship as being effective, two saw their relationship as generally effective in person but not remotely, and two felt that their coach was not effective for them in-person or remotely. Of the 12 participants in the sample that had a URM coach, nine saw their relationship as effective and met regularly remotely over the 12 months, and three saw their relationship as effective in-person but had not interacted frequently over the 12 months.

Those students who felt that coaching had been effective and who had met regularly with their coaches over 12 months, tended to emphasize the ways in which their coach’s experience enabled them to learn vicariously about how to successfully pursue an academic career. Dale’s coach was a non-URM. As we saw in his first interview, Dale characterized himself as a “trailblazer” with “no concept of the general rules of what it takes to become a PI”. In his second interview however, Dale described feeling more confident that he now understood some of the unwritten rules, or “the formula” for becoming a PI:

If your goal is to be a PI, there was just a breakdown of exactly what it is that people think. … It was just like ‘wow, there’s the formula. Everyone should know this. … What I got [that was] more than what I expected, was how to deal with the relationships, the social issues, that come up when you're dealing with your boss or other co-workers or potential employees … All these, I guess, life experiences that people pick up on, which is something that's definitely never really told to people unless you start talking. … She [the coach] was just telling her stories of how she went from different places … It’s good having someone with that experience who’s done all that to be able to tell you what they did to go through it (Dale, Hispanic Male).

Although Dale’s coach was non-URM, Dale felt he was still able to benefit from hearing her “life experiences”. This extract exemplifies how career -relevant knowledge, including knowledge of how to navigate the “social issues” (e.g. relationships with colleagues) can be made explicit, learned vicariously, and applied or adjusted to fit the learner’s circumstances and needs [14].

Alfred, as we saw in his first interview, lacked a visual goal as a result of not having seen many faculty of color. However, in his second interview, Alfred described how learning from his coach’s experiences helped to normalize and “validate” doubts over his ability to succeed:

I’m becoming more confident in that I can succeed in academia, because of the progress I’ve made and also from the Academy of getting more insight into what a career in academia entails … from our coach who talks about her experiences and how it was when she was in our position too, and just validating that these doubts or these things are normal, so it makes it easier to deal with (Alfred, Hispanic Male).

Alfred’s coach was also a successful URM scientist, with whom he was able to identify (“when she was in our position”). Alfred describes how vicariously learning about his coach’s path to an academic career had positively impacted Alfred’s self-efficacy (“I’m becoming more confident”) as well as his ability to cope with challenges and insecurities (“makes it easier to deal with”).

As we saw in their first interviews, a number of students discussed some of the challenges and incompatibilities they had experienced working with their non-URM research mentors. This included Ebony who discussed the challenges of being made to feel as though she didn’t fit the “mold” of a typical scientist–i.e. a Caucasian man with a sole focus on research. In her second interview however, Ebony described the ways in which her coach made her reconsider her identity in more positive ways:

It’s like, she [my coach] knew how I felt at certain times in my experience here–and it was small things. … Those identity type things, I always felt like I was at odds with “what are you and who are you?” And [my coach] told me it’s okay to be more than one thing and it doesn’t take away from anything else to be more than one. She [the coach] is like, “you know, there’s a box and people perceive a professor as being a certain type of way. And then she just said, ‘It’s okay to be outside the box”. It doesn’t make me less of a scientist because I want to do community work or inspire kids to do science. If anything it makes me more of a scientist. I’m proud of that now (Ebony, Black Female).

Ebony’s coach was a female URM, and had shared with Ebony and her group members many of her experiences as a former graduate student and postdoctoral researcher. In this extract, Ebony describes how she felt her coach was able to empathize with her in ways her research mentor was unable. By discussing her own experience as a URM scientist, Ebony’s coach was able to help Ebony challenge her mentor’s stereotypical perception of a PI as fitting within the “box” of a Caucasian male with a primary focus on research. Like Ebony, her coach also shared a passion for teaching and for broadening participation of minority groups within science. Hearing about how her coach had combined these elements with a successful research track record served to enhance Ebony’s perception of her self-efficacy, and sense of pride, as a scientist.

However, two students who had previously discussed the challenges of working with non-URM research mentors were randomly allocated a non-URM coach. This included Lacey who, as we saw in her first interview, felt as though no one had stood out as a mentor in regard to career development. In her second interview however, Lacey referred to her coach as a role model and described how she had overcome her own initial preference for being allocated a coach who shared the same racial background as her:

I think my academic coach has been a role model this year. … To be honest, at first I was like, “why did they assign me to her?” You know, there are other African-American coaches, and I wondered why I wasn’t assigned to them. That was my first reaction. But I definitely benefitted from having had [her] as my coach, she’s definitely helped me; can’t judge a book by its cover (Lacey, Black Female).

When asked in what ways she had benefitted from the relationship with her coach, Lacey pointed to the fact her coach was able to give “unbiased” advice and that she was “always more than willing to talk to me”.

Rosemarie, as we saw in her first interview, felt that “a lot of white men do not want to train people of color”. In her second interview however, Rosemarie discussed how she was able to “connect” to her coach, a non-URM male, leading her to challenge the view she had expressed a year prior:

I feel connected to [my coach], and, which is a paradox because I mean, honestly I was not warm to him at first, just based on my experiences with white men. So I'm glad he was able to demystify all that.

However, both of the students who felt as though the coaching they had received had not been effective were female and both had a non-URM male coach (the same coach). In addition to the coach appearing busy (as discussed earlier), one student also described how she was unable to identify with her randomly assigned coach:

It was just hard to identify with my [assigned] coach; it was hard to form any really strong connection. We have different goals entirely. I am really interested in Research I institutions and how to get to those positions. But when he gave his background he wanted time to himself, and to spend more time with his family, and didn’t want to be in a Research I institution (Carrie, Hispanic Female).

For Carrie, her inability to connect with her coach was more a result of differences in their career goals rather than differences related to their race, ethnicity or life experiences. For models to be effective and for vicarious learning experiences to be relevant, models and learners must share similar goals. Although participants were allocated a primary coach, they were also encouraged to interact with other coaches where they wished and where they felt the need. At the in-person meeting, activities such as networking social events and panel discussions encouraged communication between participants and all the Academy coaches. Carrie explained how she reached out to another coacher with whom she did connect and identify:

## Discussion

In this study, we have found that academic career coaches can serve as potential resources through which URM PhD students in the biomedical sciences are able to learn vicariously about how to pursue, and succeed within, an academic career. Coaches in this study were established life scientists with demonstrated expertise in both diversity efforts and mentoring young scientists; they were provided with additional training to help them in their coaching role.

Coaches may be particularly useful in instances where students’ mentors are unable to provide such vicarious learning opportunities, for example because the mentor is too busy to have career-related discussions with a student, or because they have, or value, a different type of academic career to the type the student hopes to achieve. Although we found that by no means all URM students are dissatisfied with their relationships with their research mentors, we found that many are not experiencing enough career-related vicarious learning opportunities. Even those students who were happy with the relationship with their research mentors, and the overall career development they had received, still felt as though they could benefit from additional career-related learning opportunities. Coaching can be an important way to address the lack of structured career development that students receive in their home training environments [6, 32].

Our findings show that although URM mentors can be seen to provide unique vicarious learning opportunities for URM PhD students, as a result of their common identity as URMs within science, non-URM mentors were just as likely to be perceived as useful. This was despite just over half the students stating, prior to the intervention, how they thought having URM faculty as mentors was important. This finding is encouraging since, by definition, URM scientists are relatively low in numbers, meaning that for any coaching initiative or program, recruiting an adequate number of URM coaches to coach URM students would be problematic. Thus, non-URM faculty with coach training can provide useful career-related vicarious learning opportunities for URM students. There was evidence in our data that coaches of color provide students with ‘visual’ examples of identifiable models who have successfully achieved their goal of an academic career. As such, interventions that seek to broaden participation of URM students in the biomedical sciences should look to include mentors or coaches of color as far as possible. However, that non-URM coaches with training were also perceived as useful is particularly encouraging since many institutions have few, or even no, URM faculty in a given department, or where existing URM faculty are already overburdened.

We found three main ways in which coaches of any race or ethnicity were useful to their students. First, quite simply, the coaches earmarked time for dedicated career guidance. Research mentors, encumbered by the many demands on their time, especially those related to their own research and grant-writing, can easily be perceived as being too busy to spend time discussing their students. In their first interviews, we found that many URM students lacked structured career development, something that supports findings from existing research [6]. However, unlike research mentors, the fact that coaches were explicitly tasked with and agreed to provide time for career guidance meant that the majority of students felt comfortable using them as a resource; a number of others also felt they were accessible, despite having no need to contact them for career guidance over the year.

A second reason why students felt their coaches were useful was because they felt both parties were able to openly and honestly discuss their experiences in academia, aided by the fact that coaches and students came from different institutions as well as usually from different fields of research. In this context, coaches and students were able to have novel types of conversation, wherein coaches discussed some of the challenges they had faced and overcome along their path to an academic career.

A third reason why students felt their coaches were useful was because their coaches provided new and alternative models for what it means to be a successful scientist. Many PhD students, irrespective of their race, ethnicity or gender, are often exposed to an implied hierarchy of academic careers or roles, with research-intensive careers at ‘research one’ institutions being assumed to be the most prestigious and thus desirable. However, recent research has shown that in the biomedical and life sciences, PhD students’ interest in a research-intensive academic career declines particularly sharply over the course of the PhD, compared to other types of science career [4]. As such, those students whose research mentors are primarily focused on research often lack adequate opportunities to learn vicariously about other types of career roles such as teaching, science communication or outreach. We found that coaches were particularly useful when they had experience in the type of career the student desired (and thus less useful where they had experience in a different type of career to the one the student desired).

We also found that the issue of intersectionality should not be ignored. Intersectionality is understood as the interrelation of multiple identity categories like race and gender. Even though this paper did not systematically set out to compare and contrast the views of URM men to URM women, we were, in our analysis, sensitized to any themes which emerged in this regard. Results published elsewhere look in more depth at the impact of the intervention in terms of gender and race/ethnicity [26]. It was notable that, in the analysis presented in this paper, that URM women were more likely to discuss significant incompatibilities in their relationship with their research mentors, compared to the URM men (beyond simply their mentor being too busy). As such, studies seeking to account for the impact of race/ethnicity on students’ career goals and graduate school experiences should also account for the ways in which gender–amongst other factors–helps to co-construct these goals and experiences. Relatedly, research on interventions that seek to broaden participation in the biomedical sciences should take into account the potentially complex ways in which race /ethnicity interact with other types of life experiences and cultural identities.

A number of limitations are important to note. Firstly, it is worth acknowledging that the sample size discussed in this paper is relatively modest, although this size is fairly common as a qualitative research sample. Future publications will look across the larger sample and explore the data quantitatively. Such quantitative analyses could further test some of the qualitative themes identified in the paper. Secondly, the study is limited by virtue of its sole focus on vicarious learning. Thus, the extent to which the vicarious learning experiences may have been affected, enhanced, mitigated or attenuated by participants’ personal performance accomplishments, social persuasions or physiological and affective states has not been addressed in this paper. Thirdly, the paper has only focused on research mentors and coaches as potential sources of vicarious learning about academic careers. Peers (including fellow graduate students and postdoctoral scientists) are also important potential sources of vicarious learning. Additionally, because of the structure of the intervention, which included group-based coaching, coaching group peers may also have been sources of vicariously learning during the intervention. Many examples of vicarious learning from peers were seen, and these questions will direct future systematic analyses and disseminations.

## Supporting Information

S1 File Interview Protocol. (DOCX)
