# Supplementary material for: Career Coaches as a Source of Vicarious Learning for Racial and Ethnic Minority PhD Students in the Biomedical Sciences: A Qualitative Study
Source: PLoS One. 2016 Jul 28;11(7):e0160038. doi: 10.1371/journal.pone.0160038 (PMC4965118; doi:10.1371/journal.pone.0160038)
Supplement: S1 File — (DOCX) [file pone.0160038.s001.docx]

**S1 File: Interview protocols^[[1]](#footnote-1)^**

**1st Interview, Prior to Start of the Coaching Intervention**

Below is a list of questions that will guide your first conversation in the research study. By having the questions in advance, you will know what to expect and have some time to think about them. During the conversation, a few more questions may be added to help understand and clarify topics that arise, and all the questions may not be covered. You are perfectly free to choose not to answer any of the questions. The purpose of the conversation is to get to know you and to learn what you are thinking about your future. All of this information will be kept confidential amongst the Principal Investigator and his team, and it will never be revealed in any way that could identify it with you. This information will not be shared with the coaches either. There are DEFINITELY no right or wrong answers or anything in particular that we want or expect to hear from you.

**Background and Experience during your PhD**

1. Tell me a bit about what it was like growing up in your family?
2. Tell me briefly about your college years and how you ended up choosing to do a PhD?
3. Was your choice of PhD program a good one for you? Why or why not?
4. What were the primary reasons you chose your PhD lab over other labs you considered? Was it an easy or difficult choice?
5. How did you arrive at your dissertation project? Did the project evolve over time or go the way you planned?
6. How would you describe the level of independence or control you had over your PhD research?
7. What do you like about doing research? What attracts you to research?
8. What don’t you like about research?
9. Now think about your experience moving into your PhD lab group. What was it like? How did you figure out how the group operated, i.e. acquiring/making sense of all the taken-for-granted, inside knowledge that you couldn’t find in a textbook. What did you do to adapt to working with that group?
10. How would you describe your ‘place’ in your PhD lab group now? Is your project a ‘high profile’ project in the lab or more of a peripheral one? Are you a leader or go-to person in the lab?
11. How much time does your PI spend with you in relation to others?
12. How well were you prepared for graduate school? What were your biggest challenges and what were you best at?
13. What do you see as your strengths and weaknesses? How do they compare to your peers?
14. Right now, do you feel like you have the balance you would like in your life? How do you see balance in terms of it getting harder or easier in the future? Why?
15. What are some of the things that have caused you the most stress in your life and in your graduate program? What do you do to reduce stress? What kinds of communities of support do you have, if any? What other resources do you draw on to address stressful issues or challenges?

**Being a Scientist**

1. When you think of a scientist, what characteristics do you think of? What does it mean to be a scientist? What does a person have to do or have in order to be a scientist?
2. To what extent is being a scientist part of your identity? [PROBE: Do you see yourself as a scientist or a part of who you are?] In what ways?
3. Do you see yourself as someone who can be successful as a scientist?
4. Do you see yourself as creative?
5. What do your family and friends think and say about your work in science and your plan to pursue science as a career?
6. How do you think your mentor sees you as a current scientist in comparison to others in your lab? How do you think your mentor sees you as a future scientist? How do you think others in your lab see you as a scientist?

**Race/ Ethnicity, Gender, SES**

As you know, we are interested in how gender, race/ ethnicity, and socio-economic status/class impact identity and how you see yourself and how others see you, particularly as it relates to your experiences in university settings.

1. Gender:
2. How has gender impacted your experiences during graduate school? What about prior to graduate school?
3. Do you think your experiences have been different than others of a different gender?
4. Has your gender impacted how you are viewed as a scientist?
5. Race/ Ethnicity/ Skin color:
6. How has skin color impacted your experiences in education and science?
7. Do you think your experiences have been different than others of a different racial or ethnic background?
8. Has your skin color impacted how you are viewed as a scientist?
9. Socioeconomic status:
10. How have socioeconomic factors impacted your experiences in education and science?
11. Do you think your experiences have been different from others of different SES?
12. Has your SES impacted how you are viewed as a scientist?
13. Of the various classifications of race, ethnicity, gender, and socioeconomic status, are any of these more important than the other(s) in terms of how you understand identity or how you identify yourself?
14. How has your gender, race, SES played a role in how you are viewed in society/in general/outside of the lab/science?
15. Have you ever felt like you are the ‘only one’ or ‘one of the only ones’? Has this in any way affected you? PROBE: Are you one of the only (PROBE whatever race/gender they are) in your lab, cohort, study groups, activities, etc.? In what way(s) and when?

**Thinking about the Future**

1. How far have you gotten in planning for a postdoc position?
2. Now let’s talk about your longer term future plans. Where would you like to be in 10-20 years’ time and why – both professionally and personally? How important is it for you to have a clear idea of where you are headed either career-wise or personally?
3. What are some of the specific steps that you know you will need to take over the next several years in order to accomplish your career goals? How confident are you that you will achieve these goals?
4. When you imagine yourself as a professor, what does this look like to you?
5. Do you think being a professor will accommodate the type of lifestyle you want to have?
6. Are there any unattractive elements in an academic career? Could any of these deter you from an academic career?
7. Do you see any major or difficult barriers, personal or professional, that you will have to overcome to achieve your career goal? How do you plan to overcome or work around these barriers?
8. What do you hear from others about academic careers? Have any messages from faculty, grad students and post docs been particularly important to you in your decision-making about careers?
9. What is your current perception of how easy or difficult it is to get a faculty position?

**Experiences with Role Models, Mentors and Others**

1. *Tell me how you define a role model and how you define a mentor.*
2. *Tell me about the role models you have had in the past or currently have. How important is it to you to have role models? Is it important that your role model has had similar life experiences to you?*
3. *Tell me about your relationship with mentors, either research mentors or others who have been mentors for you and about the mentoring you have received during graduate school.*
4. *As you finish up your program, what are you looking for from future research mentors? What will you be looking for in a postdoc lab and mentor?*
5. *Is it important that your mentors have had similar life experiences to you, pertaining to gender, race/ethnicity, socioeconomic status, sexual orientation, religion, etc.?*
6. *During the PhD so far have any of your professors, mentors or PIs shared the same racial/ethnic and/or gender background as you? Did you find yourself relating to them differently than other teachers or mentors? In what ways?*
7. *Do you have a sense of what you would like to get from coaches in the Academy, and how do you think this will compare to mentors in your PhD program and in your upcoming postdoc?*

**2st Interview, After 1 Year of the Coaching Intervention**

Below is a list of questions that will guide your 2nd conversation in the research study. By having the questions in advance, you will know what to expect and have some time to think about them. During the conversation, a few more questions may be added to help understand and clarify topics that arise, and all the questions may not be covered. You are perfectly free to choose not to answer any of the questions. The purpose of the conversation is to get an update on the previous year and learn of your plans for the future. There are DEFINITELY no right or wrong answers nor do we want or expect to hear any particular responses from you. All of this information will be kept confidential amongst the Principal Investigator and his research team, and it will never be revealed in any way that could be personally identifiable. This information will not be shared with the coaches.

**Update on Previous Year**

1. Could you start by sharing highlights of the past year? Any unexpected challenges?
2. Were there any significant changes or developments in your dissertation project?
   1. Did the project go the way you planned or undergo significant changes?
   2. Were there any changes in the level of independence or control you have over your research?
   3. Have you submitted publications and/or presented at conferences this past year?
3. Last year we asked you to describe your ‘role’ in your PhD lab group and the profile of your research. Are you a leader or go-to person in the lab? Has this role changed in the last year? Has your project move up to a ‘higher profile’ or down to a more peripheral one?
4. Describe the makeup of your lab – e.g., number of people, men vs. women, grad students, techs, postdocs, from different countries, etc. How do people get along in your lab? Have there been any problems or conflicts?
5. Are there superstars in your lab? Tell me about them.
6. Are there people who seem to be less integrated in your lab? Tell me about them.
7. What are YOUR relationships like in the lab? At this time, how connected or disconnected do you feel with the people in your lab?
8. Tell me about your relationship with your PI. How much time does your PI spend with you in relation to others?
9. Do you get direction from him/her directly or is there another person who provides that to you? If so, what is your relationship like with him or her?
10. To what degree do you actively talk with your PI about what you expect/can provide for each other? At this point, does it seem like you will be able to get the mentoring you will need and like from him or her?
    1. If not, what limitations are you seeing?
    2. Do you see other mentors or resources that could meet these potentially unmet needs, or just provide complementary guidance?
11. How about role models? Over the past year have you have you developed a closer relationship with anyone in particular? Do you go to particular mentors/advisors depending on the type of advice you are seeking (i.e., strictly professional, career-life choices, etc.)?
12. What are some of the things that have caused you the most stress in your life and in your graduate program during the last year? What did you do to reduce stress? What kinds of communities of support do you have, if any? What other resources do you draw on to address stressful issues or challenges?

**Graduation & Future Plans**

1. In terms of long-term career goals, has your vision of where you see yourself in 10-15 years, either professionally or personally, changed over the past year?
2. Have any ‘messages’ you heard from faculty, grad students and post docs been particularly important to you in your decision-making about careers over this past year?
3. If you are currently in a postdoc position:
   1. How long have you been in your new lab? What is your experience like so far?
   2. Why did you choose to join this particular lab?

For questions 17-19, if you have already graduated or defended your dissertation, please answer them with respect to the time between last summer and completion of your degree – i.e. the finishing up time. If you are still working to complete your degree, please answer them with respect to your progression toward completion since last summer.

1. Progressing towards graduation: what were/are your expectations and the expectations of your PI for:
   1. Time for writing (e.g. diminished expectations for research productivity, dedicated time to write)
   2. Support for writing (e.g. feedback from dissertation advisor and/or committee members)
2. What were/are your immediate plans after graduation? How did you plan/are you planning for a postdoc or other position?
3. Did/does your PI know the details of your plans and goals? Did/do you and your PI see eye-to-eye on your future plans?
4. As you continue your career, what are you looking for from a research mentor?
5. To what extent does/did your relationship with your mentor(s) inform or contribute to how you see yourself as a mentor in the future?
6. Are you conscious of how any particular strengths or weaknesses will affect your experience post-graduation/in the job market? Have you made any conscious effort(s) to work on or enhance particular skills over the last year?
7. Do you see any major or difficult barriers, personal or professional, that you will have to overcome to achieve your career goal? How do you plan to overcome or work around these barriers?
8. What do you hear from others about academic careers? What is your current perception of how easy or difficult it is to get a faculty position? Are there any unattractive elements in an academic career (research or teaching-focused) that could particularly influence your decision not to pursue it?

**Being a Scientist**

1. Have your views changed over the past year in any way in terms of what it means to be a scientist? Have your views changed in terms of how you see YOURSELF as a scientist? What about how others see you as a scientist?
2. How do you think your PI sees you as a current scientist in comparison to others in your lab? Your potential as a scientist in the future? How do you think your PI and/or other mentors sees you and/or portrays you to others as a scientist? How do you think others in your lab see you as a scientist?

**Race/ Ethnicity, Gender, SES**

As you know, we are learning about how gender, race/ ethnicity, and socio-economic status/class impact identity and how you see yourself and how others see you. Reflecting first on ONLY your experiences in this past year:

1. Gender:
2. Has your gender impacted your experiences during this last year? If so, how?
3. Do you think your experiences have been different than others of a different gender?
4. Has your gender impacted how you are viewed as a scientist?
5. How do you think your gender will impact your experiences in the next phase of your career?
6. Race/ Ethnicity/ Skin color:
7. Has skin color impacted your experiences? If so, how?
8. Do you think your experiences have been different than others of a different racial or ethnic background?
9. Has your skin color impacted how you are viewed as a scientist?
10. How do you think your race/ethnicity/skin color will impact your experiences in the next phase of your career?
11. Socioeconomic status:
12. Have socioeconomic factors impacted your experiences? If so, how?
13. Do you think your experiences have been different from others of different SES?
14. Has your SES impacted how you are viewed as a scientist?
15. How do you think your SES will impact your experiences in the next phase of your career?
16. Reflect on your closest relationships with any mentors or role models. Do you notice if they share similar life experiences to you, pertaining to gender, race/ethnicity, socioeconomic status, sexual orientation, religion, etc.? Do you find yourself relating to them differently than other teachers or mentors? In what ways?
17. Of the various classifications of race, ethnicity, gender, and socioeconomic status, are any of these more important than the other(s) in terms of how you understand identity or how you identify yourself?
18. Have you felt like you are the ‘only one’ or ’one of the only ones’ over the past year? Has this in any way affected you?
19. Has your participation in the Academy caused you to reflect on or interpret differently your experiences BEFORE starting graduate school, in terms of gender, race/ethnicity or socio-economic status?

**Coaches and the Academy:**

1. *We are really interested in gathering your input about how the Academy and coaching model worked and what elements were most effective and what needs to be changed. Do you have any thoughts or suggestions?*

*After you left the Academy, what were your expectations of the coaches during the upcoming year? Were your expectations met? Was your coach/coaching group useful during the year? In what ways? If you didn’t reach out to your coach, why not?*

*Can you tell me about a particularly memorable moment from your interaction with your coach?*

Did you ever turn to other Academy students for help with a particular issue? Tell me about it.

I’ve read your answers in the survey about whether you’ve used the social science theories in the past year. There are a few points that I would like you to expand on.

This question is different as we are using this as feedback for coaches-they are interested in continually improving their skills as coaches. What could the coach have done differently to make the process more useful for yourself and/or the group as a whole? Is there anything that got in the way of the coach’s effectiveness for you and/or the group?

1. Findings for this paper were taken from an analysis of the interviews as a whole, although those questions that were a particular focus of this paper’s analysis are underlined and italicized. [↑](#footnote-ref-1)
